# Global identification, structural analysis and expression characterization of bHLH transcription factors in wheat

**DOI:** 10.1186/s12870-017-1038-y

**Published:** 2017-05-30

**Authors:** Xiao-Jiang Guo, Ji-Rui Wang

**Affiliations:** Triticeae Research Institute, Sichuan Agricultural University, Chengdu, 611130 China

**Keywords:** bHLH, Transcription factor, RNA-Seq, Expression pattern, Wheat

## Abstract

**Background:**

Basic helix-loop-helix (bHLH) transcription factors (TFs), which are widely distributed in eukaryotic organisms, play crucial roles in plant development. However, no comprehensive analysis of the bHLH family in wheat (*Triticum aestivum* L.) has been undertaken previously.

**Results:**

In this study, 225 bHLH TFs predicted from wheat using genomic and RNA sequencing data were subjected to identification, classification, phylogenetic reconstruction, conserved motif characterization, chromosomal distribution determination and expression pattern analysis. One basic region, two helix regions and one loop region were found to be conserved in wheat bHLH TFs. The bHLH proteins could be separated into four categories based on sequences in their basic regions. Neighbor-joining-based phylogenetic analysis of conserved bHLH domains from wheat, *Arabidopsis* and rice identified 26 subfamilies of bHLH TFs, of which 23 were found in wheat. A total of 82 wheat bHLH genes had orthologs in *Arabidopsis* (27 TFs), rice (28 TFs) and both of them (27 TFs). Seven tissue-specific bHLH TF clusters were identified according to their expression patterns in endosperm, aleurone, seedlings, heading-stage spikes, flag leaves, shoots and roots. Expression levels of six endosperm-specifically expressed TFs measured by qPCR and RNA-seq showed a good correlation.

**Conclusion:**

The 225 bHLH transcription factors identified from wheat could be classed into 23 subfamilies, and those members from the same subfamily with similar sequence motifs generally have similar expression patterns.

**Electronic supplementary material:**

The online version of this article (doi:10.1186/s12870-017-1038-y) contains supplementary material, which is available to authorized users.

## Background

Transcription factors (TFs) regulate gene expression in different tissues during various developmental stages in plants, thereby controlling agronomic and economic qualities of crops. TFs usually contain two different functional domains that are involved in DNA binding and protein dimerization activities regulated by several mechanisms, including differential dimer formation [1, 2]. Basic helix-loop-helix (bHLH) proteins constitute one of the largest TF families and are widely distributed across eukaryotic kingdoms [1, 3]. bHLH proteins are usually classified into different subfamilies and subgroups based on sequences of their DNA-binding or protein-binding domains, which are highly conserved among species [3–5]. The bHLH domain generally contains approximately 60 amino acids and possesses two functionally distinct regions, a basic region and a helix-loop-helix (HLH) region [3, 4]. The basic region, located at the N-terminus along with a DNA-binding motif, comprises about 15 amino acids—typically including six basic residues [4, 6]. The HLH region, containing two amphipathic α-helices separated by a loop region of variable length or sequence, acts as a dimerization domain and allows the formation of homodimers or heterodimers [7–9]. In all bHLH motifs, 19 amino acids have been found to be highly conserved in organisms ranging from yeast to mammals [10, 11]. Other than these conserved domains, bHLH proteins exhibit considerable sequence divergence. In addition, the nucleotides flanking the core element may play a role in binding specificity [4, 12].

According to their phylogenetic relationships and functional properties, DNA-binding motifs of known bHLH TFs have been divided into six main groups (A to F) containing 45 subfamilies [3, 13, 14]. Group A contains 22 subfamilies, such as Atonal, D, Delilah, dHand, E12, Hen, Lyl, MyoD and Twist, that can bind to E-boxes (5′-CANNTG-3′) of TF-regulated genes. Group B comprises 12 subfamilies, including Max, Myc, MITF, SREBP and USF, which are able to bind to the G-box (5′-CACGTG-3′). Group C consists of seven subfamilies that possess both a bHLH domain as well as a protein–protein interaction region (the PAS domain) binding to ACGTG or GCGTG sequences. Group D has only one subfamily, whose members all lack a basic domain. Because these TFs can form heterodimers with bHLH proteins from group A, they are functionally related to typical bHLH proteins [15]. Group E contains two subfamilies with Pro or Gly residues in the basic region that bind preferentially to typical N-box sequences (CACGCG or CACGAG) [16]. Compared with the other groups, group F proteins have divergent sequences and contain an additional domain for dimerization and DNA binding [3, 16, 17].

Plant bHLH proteins have been well characterized only in *Arabidopsis*, rice (*Oryza sativa* L.), Chinese cabbage (*Brassica rapa* L.), tomato (*Solanum lycopersicum* L.), *Nicotiana tabacum* and *Salvia miltiorrhiza* [6, 18–21], where they have been divided into 21, 22, 24, 23, 24 and 25 subfamilies, respectively. Phylogenetic analysis has shown that plant bHLH proteins comprise 26 subfamilies, 20 of which were present in the common ancestors of extant mosses and vascular plants [22]. Investigations conducted in different plant species have revealed the wide and diverse array of biological processes in which these proteins are involved [23–25].

In this study, we investigated bHLH TFs in wheat (*Triticum aestivum* L.) through a comparative genomic analysis. The aims of this study were as follows: (1) genome-wide characterization of bHLH TFs from wheat; (2) prediction of conserved residues of wheat bHLH TF genes; (3) analysis of phylogenetic relationships of wheat bHLH TFs and those of other plants; (4) chromosomal localization of bHLH TFs and identification of syntenic relationships with rice and *Arabidopsis*; and (5) expression profiling of bHLH TFs by transcriptomic sequence, expression array and qPCR analyses.

## Methods

### Plant materials

Chinese Spring (landrace), CM32 (cultivar) and a synthetic hexaploid wheat SHW-L1 were used for expression pattern analysis of wheat bHLH genes at different development stages according to qPCR. All materials were offered by Triticeae Institute of Sichuan Agriculture University. Root, stem, leaf and seed samples were collected from materials growing in greenhouse under a 16/8-h day-night photoperiod at 18 °C and 55–60% relative humidity in July to September, 2016. For Chinese Spring, samples included leaves and roots from seedlings at the five-leaf stage, 1-week-old whole seedlings, and germinating seeds at 6, 12, 24, 36 and 48 h after imbibition (HAI). Samples of CM32 and synthetic hexaploid wheat consisted of leaves, roots and stems at the seedling stage and seeds at 5, 10, 15, 20, 25 and 30 days post-anthesis (DPA). All samples were snap-frozen in liquid nitrogen and stored at −80 °C. Total RNA was isolated with Trizol reagent (Biofit Biotechnology, Chengdu, China) according to the manufacturer’s instructions. The RNA samples were stored at −80 °C. First-strand cDNA synthesis was carried out using a Prime-Script TM RT reagent kit (Takara Biotechnology, Dalian, China) according to the manufacturer’s instructions.

### RNA sequencing (RNA-seq) and expression array datasets for identification of bHLH TFs

Wheat bHLH proteins identified from previous studies were downloaded from WheatTFDB (http://xms.sicau.edu.cn/wheatTFDB/) and PlantTFDB V3.0 (http://planttfdb.cbi.pku.edu.cn/) databases [26, 27]. bHLH protein sequences of rice and *Arabidopsis* were obtained according to Pires and Dolan (2010). Housekeeping genes as expression references were obtained from NCBI and used to normalize the expression of bHLH proteins in wheat (Additional file 1: Table S1). For use in further analyses, we selected 104 RNA-seq (transcript) data files with tissue or organ information at different wheat developmental stages from over 700 wheat RNA-Seq data files currently available in the SRA database (http://www.ncbi.nlm.nih.gov/sra/?term=) (Additional file 2: Table S2). Sequences of wheat cultivar Chinese Spring from the Chromosome Survey Sequence data file popseq.30.dna (ftp://ftp.ensemblgenomes.org/pub/plants/release-30/fasta/) were used to analyze the chromosomal locations of bHLH TFs [28]. Homologous genes of wbHLH were obtained from IWGSC (http://www.wheatgenome.org/) and TGAC (http://browser.tgac.ac.uk/) databases. The 61-kb wheat genome array and expression profiling data in PLEXdb (http://www.plexdb.org/index.php) were used to identify the expression of bHLH TFs.

### bHLH domain structure prediction

For bHLH domain structure prediction, bowtie2 (http://sourceforge.net/projects/bowtie-bio/files/bowtie2/) was first applied to map RNA-Seq reads to the wheat genome sequences. Cufflinks (http://www.sihua.us/Cufflinks.htm) was then used to assemble the mapped reads into the 104 RNA-seq data files. The program cd-hit-est. was used to cluster redundant sequences (−c 0.95 -n 8), and the ESTate package (http://weizhongli-lab.org/cd-hit/) was used to translate nucleic acid sequences into protein sequences. Redundant protein sequences were filtered out using the cd-hit program (−c 0.95 -n 5). HMMER v3.0 (http://hmmer.janelia.org/) was used to predict wheat bHLH TFs.

### Phylogenetic analysis and identification of conserved motifs

Protein sequences were screened against the Pfam database to identify domains of bHLH TFs. ClustalX 2.0 software was used to construct a neighbor-joining tree of wheat bHLH protein key domain sequences [29]. The identified bHLH domains were aligned using ClustalX 2.0 with default settings [30]. To identify conserved motifs in wheat bHLH proteins, MEME v4.11.0 (http://meme-suite.org/tools/meme) was used with default settings, except that the optimum motif width was set to ≥10 and ≤100 and the maximum number of motifs was set to identify 10 motifs. MEME was also used to search for conserved motifs in the full amino acid sequences of bHLH.

### Gene ontology and enrichment analysis

Annotation and identification of enriched pathways was performed using the KOBAS 2.0 analysis toolkit (http://kobas.cbi.pku.edu.cn/index.php) [31]. The WEGO online tool (http://wego.genomics.org.cn/cgi-bin/wego/index.pl) was used to perform gene ontology enrichment analysis of identified bHLH genes [32]. The Blast program was used to compare the data from *Arabidopsis* and rice, with genes subsequently removed on the basis of the following criteria: aligned amino acid sequence length > 60 (because bHLH proteins contain about 60 conserved amino acid residues; William, et al. 1999) and % identity >80%. An interaction network associated with *Arabidopsis* orthologs of bHLH genes in wheat was constructed using the *Arabidopsis* Interaction Viewer and Cytoscape 3.3 software [33]. The STRING v10.0 database (http://string-db.org/) was used to identify protein–protein interactions of bHLH proteins.

### Chromosomal locations and collinearity of bHLH genes

The position of each bHLH gene on the 21 wheat chromosomes was determined by mapping bHLH gene sequences to Chinese Spring wheat Chromosome Survey sequences using Blat and Blast programs. The positions of *Arabidopsis* and rice bHLH genes were also mapped. The identified positions were then marked on the chromosomes using the Circos tool [34].

### Analysis of bHLH gene expression in wheat

TopHat (http://ccb.jhu.edu/software/tophat/manual.shtml) and Cufflinks were used to analyze the expression of bHLH family genes in wheat. TopHat13 (http://tophat.cbcb.umd.edu/) is a program that aligns reads to the genome and discovers transcript splice sites, while Cufflinks8 (http://cufflinks.cbcb.umd.edu/) compares the generated map from TopHat13 against the genome to assemble the reads into transcripts [35].

RNA-seq reads were first mapped to wheat bHLH family genes using TopHat. The RNA-seq reads for each biological replicate were mapped independently. These mapped reads were input into Cufflinks, which produced one file of assembled transcribed fragments per replicate. The assembly files were then merged with the reference transcriptome annotation into a unified annotation for further analysis. This merged annotation was quantified under each condition by Cuffdiff, which produced expression data as a set of tabular files. The tabular files were indexed and visualized with CummeRbund to facilitate exploration of genes identified by Cuffdiff as differentially expressed. After generating expression data in terms of FPKM (fragments per kilobase of transcript per million fragments mapped) [35], we divided expression levels of bHLH genes by the average expression of the reference genes (Additional file 3: Table S3). We then combined the data using the ‘scale’ function in R (https://www.r-project.org/) and used the pheatmap tool in R to draw a heat map of bHLH family gene expression levels in wheat. Finally, bHLH TF expression data were obtained by combining GeneChip and expression profiling data from PLEXdb (http://www.plexdb.org/index.php).

### Expression confirmation by qPCR

Specific primers were designed according to bHLH gene sequences using Primer 5.0 software (Premier Biosoft International, Palo Alto, CA, USA) (Additional file 4: Table S4). Reference genes were used as an internal control to normalize the expression level of the target gene among different samples [36]. qPCR amplification was performed with SYBR Premix Ex *Taq* (2×) (Takara) using a MyiQ Single Color Real-Time PCR detection system (Bio-Rad, Hercules, CA, USA).

## Results

### Characterization of bHLH TFs in wheat

**Fig. 1:**
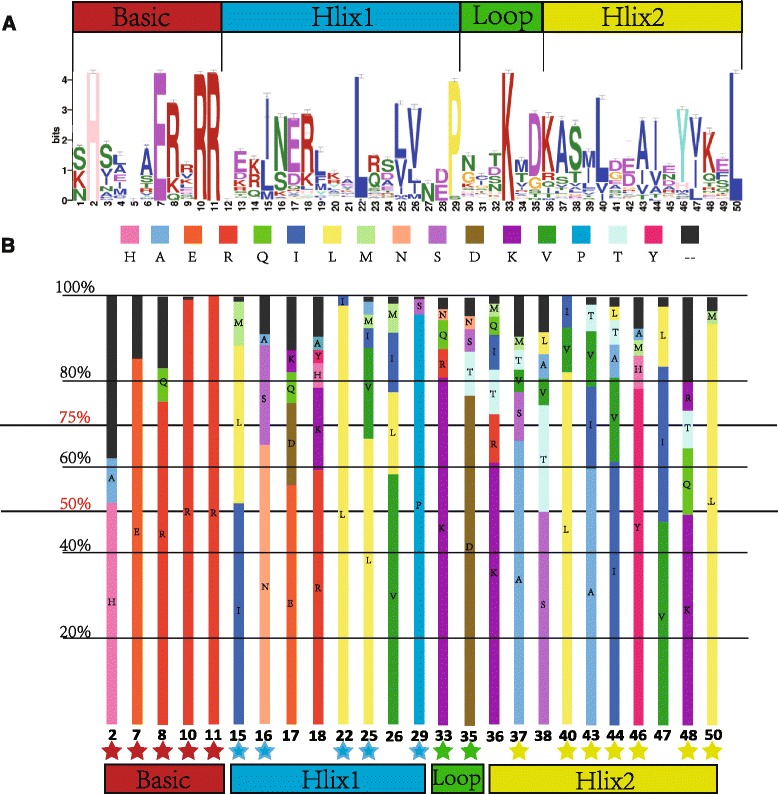
Characterization and distribution of bHLH domains. **a** Sequence logo of the bHLH domain generated in MEME. Amino acids important for dimerization of the helix-loop-helix domain are indicated by stars. **b** Distribution of amino acids in the bHLH consensus motif of wheat bHLH transcription factors. The numbers at the bottom are positions of the residues in the alignment

In this study, we obtained 225 putative bHLH TFs with typical conserved sequences (Additional files 5, 6: Tables S5, S6), among which 38 bHLH TF genes were common to this study and other databases (plantTFDB and WheatTFDB). And 121, 33 and 36 bHLH TFs were specific in this study, plantTFDB and wheatTFDB, respectively (Additional file 7: Figure S1). To characterize sequence features of wheat bHLH TFs, a multiple sequence alignment based on protein sequences of these 225 TFs was generated. Four typical conserved bHLH regions, namely, one basic region, two helix regions and one loop region, were detected in wheat bHLH TFs (Fig. 1, Additional file 7: Figure S1). The first helix region had five conserved residues (Ile-15, Leu-16, Leu-22, Val-25 and Pro-29), while the second helix region comprised seven (Ala-37, Leu-40, Ala-43, Ile-44, Tyr-46, Lys-48 and Lys-50). The loop region possessed only two conserved residues (Lys-33 and Asp-35). The basic region contained five conserved residues (His-2, Glu-7, Arg-8, Arg-10 and Arg-11). Although the basic region was the most conserved bHLH TF region, 19 of the 225 proteins did not contain it. The loop region was found to be the most divergent in terms of both length and amino acid composition (Additional file 8: Figure S2). According to the sequence alignment data, 19 conserved amino acid residues were found in more than 100 wheat bHLH TFs (Fig. 1). Among these residues, Glu-7, Arg-8, Arg-10, Arg-11, Leu-22, Pro-29, Lys-33, Asp-35, Leu-40, Tyr-46 and Leu-50 were found to be conserved in approximately 75% of TFs.

Using the criteria of Massari and Murre (2000) (40), the 225 bHLH proteins were separated into four categories according to sequence profiles in their basic regions: 133 E/G-box binding, 45 E-box binding, 28 other binding and 19 non-binding bHLHs. According to the number of amino acid residues in the basic region, wheat bHLH TFs were separated into two major groups, namely, groups with (206) or without (19) DNA-binding regions. Among the 206 TFs with DNA-binding regions, 133 TFs had both G-box and E-box recognition sites, 45 TFs had E-box recognition sites only, 28 TFs had other binding sites and 19 TFs had no binding sites.

### Phylogenetic analysis of wheat bHLH TFs

**Fig. 2:**
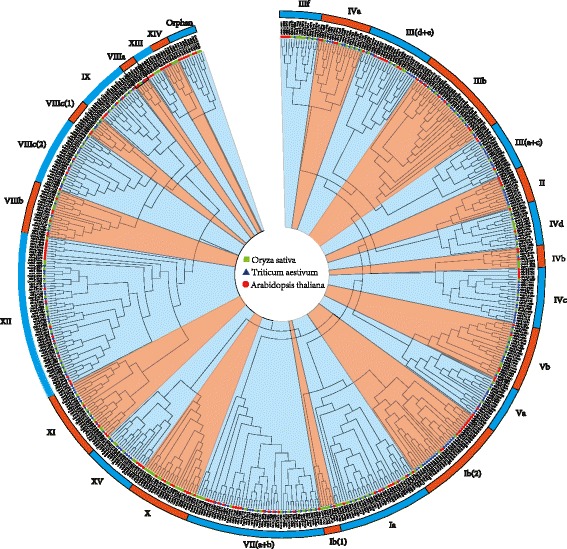
Phylogenetic tree of bHLH transcription factor domains in wheat, *Arabidopsis* and rice. The phylogenetic tree was constructed by the neighbor-joining method using ClustalX2 and MEGA6 software. Bootstrapping with 1000 replicates was used to assess the statistical reliability of nodes in the tree

**Fig. 3:**
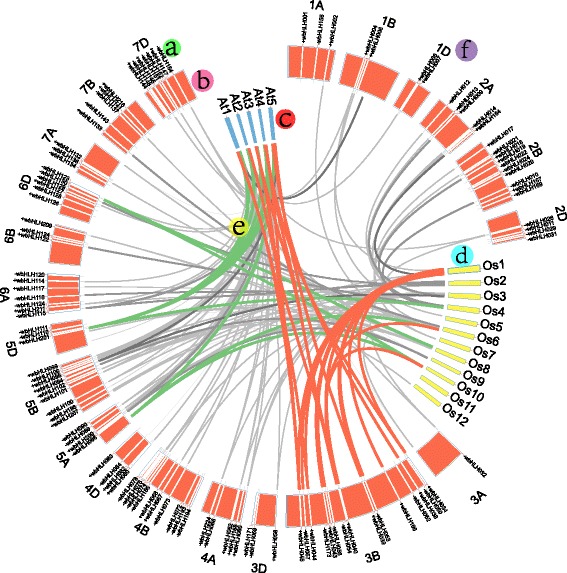
Chromosomal distribution and syntenic relationships of the bHLH transcription factor family. bHLH gene locations, chromosomes and chromosome complements are indicated by lowercase letters (**a**, **b** and **f**), respectively. The chromosomes of *Arabidopsis* and rice are indicated by lowercase letters (**c** and **d**), respectively. Wheat bHLH transcription factor genes in synteny with regions in *Arabidopsis* and rice are indicated by lowercase letter (**e**)

To assess the evolutionary relationships of bHLH TFs in plants, a neighbor-joining phylogenetic tree was generated using conserved bHLH domains from wheat, *Arabidopsis* and rice (Fig. 2). Twenty-six subfamilies were identified, consistent with results previously obtained from *Arabidopsis* and rice. To label these wheat subfamilies, we therefore adopted the *Arabidopsis* bHLH group nomenclature proposed by Pires and Dolan (2010). The 225 wheat bHLH TFs were grouped into 23 of the 26 subfamilies (Fig. 3), with subfamilies XIII, XIV and X the only ones lacking bHLH TFs from wheat. The MEME program was used to identify conserved motifs of TFs in each bHLH subfamily. Fifteen conserved motifs were identified and designated as motifs 1 through 15 (Additional file 9: Figure S3). Most bHLH TFs in the same subfamily clearly had similar motifs (Additional file 9: Figure S3).

### Chromosomal distribution and synteny analysis

Because the wheat genome has not yet been fully sequenced, only 168 of the 225 wheat bHLH genes were successfully mapped to the wheat survey sequence (Fig. 3); others were mapped onto the wheat scaffolds (Additional file 10: Table S7). The largest number of these bHLH TFs were distributed on wheat chromosome 3B, while chromosomes 1A and 1D contained the least (Additional file 11: Table S8).

A total of 82 wheat bHLH genes had orthologs in *Arabidopsis* (27 TFs), rice (28 TFs) and both of them (27 TFs) (Additional file 12: Table S9, Fig. 3). For example, wbHLH006 on chromosome 1B, is orthologous to genes on *Arabidopsis* (AtbHLH008, PIF3) chromosome 1 and rice (OsbHLH106) chromosome 5; wbHLH026 on chromosome 2D, is orthologous to genes on *Arabidopsis* (AtbHLH015, PIL5) chromosome 2 and rice (OsbHLH152) chromosome 3; and wbHLH095 on chromosome 1B, is orthologous only to genes on *Arabidopsis* (AtbHLH006, MYC2) chromosome 1 (Fig. 3).

### Gene ontology and enrichment analysis

**Fig. 4:**
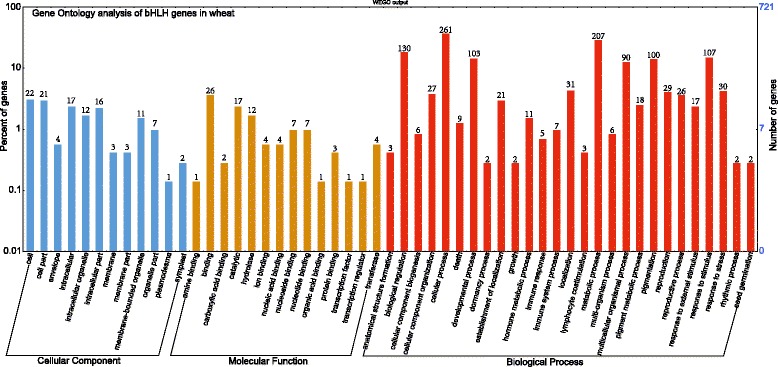
Gene ontology analysis of bHLH genes. The results are classified into three main categories: cellular component, molecular function and biological process. The y-axis indicates the percentage of genes in a given category, while numbers above bars represent the number of genes in that category

Functional annotation of the 225 bHLH TFs revealed their association with a range of cellular component, molecular function and biological process categories (Fig. 4). The majority of functions were biological processes, such as cellular process (261 terms), metabolic process (207), biological regulation (130), response to stimulus (107), developmental process (103), pigmentation (100), multicellular organismal process (90), response to stress (30), hormone metabolic process (11), dormancy process (2) and seed germination (2) (Fig. 4). The most highly enriched molecular function categories were related to binding (26), catalysis (17), hydrolase (12), nucleoside binding (7), nucleotide binding (7), ion binding (4), transferase (4) and protein binding (3) (Fig. 4). Most bHLH genes assigned to the binding category were involved in binding to DNA, proteins and ions. The cellular component category included terms related to cell (22), cell part (21), envelope (4), intracellular (17), intracellular organelle (12), intracellular part (16) and membrane (3) (Fig. 4).

On the basis of these annotations, only 20 bHLH TFs from 82 orthologous wbHLH proteins of *Arabidopsis* and rice could be annotated in eight protein–protein interaction networks and identified in the STRING database (Additional files 12, 13: Table S9, Figure S4). Two Kyoto Encyclopedia of Genes and Genomes (KEGG) pathways involving bHLH proteins were also detected, with proteins wbHLH006 (PIF3) and wbHLH095 (MYC2) involved in the plant hormone signal transduction pathway (Additional file 14: Figure S5) and wbHLH006 (PIF3) regulating light signal transduction in the circadian rhythm pathway (Additional file 15: Figure S6). The same KEGG pathways were also obtained when we annotated wbHLH TF proteins in sorghum and maize (Additional files 16, 17, 18, 19: Figures S7–10).

### Differential expression of bHLH genes in various tissues

**Fig. 5:**
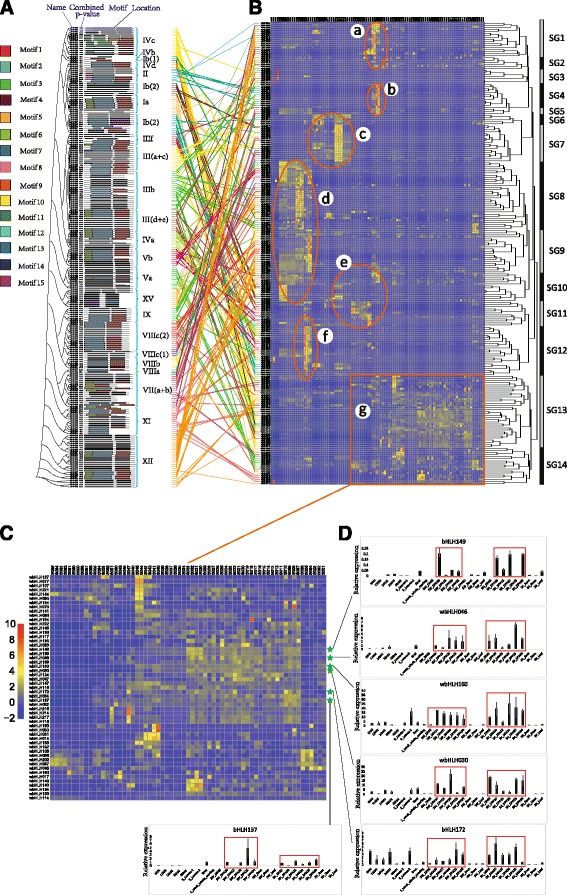
Heat map of expression levels of wheat bHLH transcription factor family genes at different growth periods in different tissues and organs. The lines link gene structures and expression levels. Each subfamily is represented by a different color. **a** Grouping of 225 bHLH genes into 24 subfamilies according to gene structure. **b** Clustering of bHLH gene expression patterns into 14 subgroups (SGs). Seven blocks (**a**–**g**) correspond to higher expression levels. **c** Enlarged map section showing details of bHLH gene expression of block g as an example. **d** These expression profile data were obtained using qPCR. The ‘scale’ function in R was used to normalize relative expression values. The heat map was generated using the Pheatmap package of R. FPKM values were normalized using the ‘scale’ function in R

The expression patterns of 225 wheat bHLH TFs were characterized at different growth stages in different tissues and organs using the 104 RNA-seq data files (Additional file 3: Table S3; Fig. 5b). Seven obvious blocks (a–g) of bHLH TFs with significant altered expression levels in endosperm, aleurone, seedlings, heading-stage spikes, flag leaves, shoots and roots were detected. Blocks a–d and f, but not blocks e and g, showed obvious tissue-specific expression (Fig. 5b). The bHLH genes contained in blocks a and b were mainly expressed in spikes at the heading stage and in anthers at the uninucleate pollen stage. Most bHLH genes in block c displayed higher expression levels in shoots than in leaves (including Zadoks-13 stage leaves, 15-DPA flag leaves, 25-DPA flag leaves, 30-DPA flag leaves, heading-stage flag leaves and first-flowering-date flag leaves). The bHLH genes in block d were mainly expressed in 1-week-old roots and leaves. We found that the bHLH genes in block e exhibited higher expression levels in aleurone, heading-stage spikes, *Fusarium graminearum* inoculated spikelets and non-inoculated spikelets, and 1-week-old leaves. The bHLH genes in block f also showed high expression levels in 1-week-old leaves. Although bHLH genes in block g showed nonspecific expression in endosperm, aleurone, inner pericarps, outer pericarps, transfer cells, caryopses, and heading-stage spikes, all of these tissues are associated with seed.

As an example, the enlarged area of the heat map in Fig. 5c shows the high expression of bHLH genes in subgroup SG12 (Fig. 5c). Expression levels of the selected 24 bHLH TFs gradually increased in leaves under salt stress from 6 to 48 h, whereas no significant variance in expression was detected among non-salt-stressed samples from leaves, roots, seedlings, endosperm, aleurone, spikelets and heading-stage flag leaves. Genes from the same subfamily had obviously similar expression patterns. For example, bHLH073, bHLH063, bHLH080, bHLH195 and bHLH070 of subfamily IVa (Fig. 5a, c) exhibited similar expression patterns in all samples, as did bHLH035, bHLH037, bHLH036, bHLH034, bHLH033, bHLH049 and bHLH048 of subfamily Ib(2) (Fig. 5a, c). However, some other genes of the same subfamily, such as bHLH058, bHLH175 and bHLH071 of subfamily IB(2), displayed contrasting expression patterns (Fig. 5a, c). Moreover, some genes from different subfamilies showed similar expression patterns, such as bHLH168 (IIf subfamily), bHLH044 (IX subfamily) and bHLH040 [III (d + e) subfamily].

Considering our results from another perspective, we present the XII subfamily as an example. This subfamily exhibited three main expression patterns corresponding to different subgroups on the heat map. First, the five bHLH TFs (wbHLH094, wbHLH206, wbHLH143, wbHLH138 and wbHLH151) in SG1 all had the same expression pattern (Fig. 5a, b). Second, the expression patterns of nine bHLH TFs (wbHLH181, wbHLH177, wbHLH109, wbHLH200, wbHLH027, wbHLH011, wbHLH018, wbHLH057 and wbHLH103) in SG9 were also identical to one another (Fig. 5a, b). Finally, wbHLH136, wbHLH145, wbHLH222 and wbHLH137 in SG13 all had different expression patterns (Fig. 5a, b).

We obtained expression data by combining GeneChip and expression profiling data from PLEXdb. Block b contained two subgroups (SG4 and SG5); block c did likewise (SG6 and SG7) (Fig. 5b). The bHLH genes of SG4 and SG5, except for wbHLH183, displayed relatively high expression levels in anthers before anthesis (Additional file 20: Figure S11). Most genes of SG6 and SG7 also showed relatively high expression levels in leaves at this stage (Additional file 21: Figure S12). We compared the expression profiles of bHLH TFs in block g (SG13 and SG14) by RNA-seq and qPCR. The expression levels of these endosperm-specifically TFs were well correlated between the two methods (*r* = 0.58–0.87, *P* < 0.05) (Additional file 22: Table S10).

## Discussion

### Characteristics of bHLH family genes in wheat

In this study, we systematically predicted and identified 225 bHLH TF members in the wheat genome. Based on the characterization of bHLH TFs according to expressed sequence tag data (plantTFDB and wheatTFDB), we used RNA-seq data to predict bHLH TFs that resulted in 121 bHLH TFs newly identified and localized on wheat genome survey sequences (Additional file 23: Table S11). This success can be attributed to the fact that RNA-seq achieves single-nucleotide resolution, has a substantially higher dynamic range relative to expressed sequence tag sequencing, and allows reliable identification of rare transcripts and alternative splicing [37–40].

The bHLH proteins were separated into four categories based on sequence profiles of their basic regions. Consistent with previous studies [6, 8, 19–21], most of these proteins contained 19 conserved amino acid sites (Fig. 1a, b). Leu-22 in the bHLH Hlix1 region is the most conserved amino acid residue in plants, including in *Arabidopsis*, Chinese cabbage, tomato and rice [19, 20]. This residue, which may be extremely important for promoting the formation of dimerization among bHLH proteins, is less conserved in dicots than monocots. Interestingly, we found that Leu-22 was mutated to Ile-22 in only five wheat bHLH proteins (wbHLH043, wbHLH051, wbHLH090, wbHLH186 and wbHLH225) and has been lost in wbHLH106 (Additional file 8: Figure S2).

The genome of hexaploid wheat contains 16,000 Mb of DNA originating from the natural hybridization of three genomes: A, B and D [41]. On the basis of the characteristics of bHLH conserved regions [3–5], we attempted to locate homologs of putative bHLH genes in the wheat genome database (IWGSC; http://www.wheatgenome.org/) and TGAC (http://browser.tgac.ac.uk/). Among the 225 identified wbHLH TFs, homologs of 28 appeared to be absent from either A, B or D genomes, while 4 were not located on two genomes (Additional file 24: Table S12). Two possible reasons for their absence are gene loss during evolution [42] and the parameters used for bioinformatics analyses.

Phylogenetic analysis of 225 distinct protein sequences clearly divided the bHLH genes into 23 subfamilies. This classification was further supported by the results of gene structure and motif analyses (Additional file 9: Figure S3). The topology of our phylogenetic tree, constructed from bHLH genes of three species (wheat, rice and *Arabidopsis*), is generally consistent with a previously published tree derived from *Arabidopsis* and rice. In earlier studies, the number of bHLH TF family members has varied among species, with 158 bHLH TFs identified in *Arabidopsis* and 289 in soybean, 289 in maize, 173 in rice, 230 in Chinese cabbage and 127 in potato [19, 20, 22]. This abundance indicates that the bHLH TF family has an essential role in growth and development of wheat and other plants.

### Potential roles of bHLH genes in wheat

bHLH genes have been extensively studied and found to be involved in a wide variety of plant processes. For instance, bHLH TFs are involved in stress response, plant development, metabolite biosynthesis and trait development. Specific examples include response to cold (AtbHLH116, ICE1 and AtbHLH003) and heat (AtbHLH009), abscisic acid and jasmonic acid responses as well as light-signaling pathways (AtbHLH006, MYC2), root hair formation (AtbHLH083), anther development (AtbHLH021) and axillary meristem generation (OsbHLH123) [22]. The functional annotation of wheat bHLH genes in this study revealed their responsiveness to abiotic stress and hormone signal transduction (Fig. 4). Our protein–protein interaction network contained 20 wbHLH proteins with significant homology to *Arabidopsis* that had associations with abiotic stress and endogenous hormone signal transduction. For example, wbHLH221 (*SPT*) expression is transcriptionally induced by light and cold in imbibed seeds [43], wbHLH012 (*PIL6*) is implicated in light-signal transduction [44] and *PIL5* is a phytochrome-interacting bHLH TF that negative regulates seed germination [45] (Additional file 13: Figure S4). Wheat TF proteins wbHLH006 and wbHLH095, which are respectively close orthologs of *Arabidopsis* MYC2 and PIF3, were found to be involved in two KEGG pathways in *Arabidopsis* but not in rice. wbHLH133 (TabHLH1) is able to improve tolerance to phosphorus and nitrogen deprivation via regulation of nutrient transporter gene transcription and reactive oxygen species homeostasis [46]. wbHLH002 (TabHLH060) has been demonstrated to enhance the susceptibility of transgenic *Arabidopsis* to *Pseudomonas syringae* [47]. wbHLH010 (*TaMYC1*) regulates anthocyanin synthesis in wheat pericarps [48]. wbHLH046 (*ICE1*) enhances freezing tolerance, while wbHLH172 (CA599618) has been found to be affected by salt stress in a tolerant wheat cultivar [49, 50].

### Expression patterns of bHLH genes imply cooperative action and functional differentiation

bHLH TF genes from the same subfamily had similar expression patterns, with some genes belonging to different subfamilies showing similar expression patterns (Fig. 5a, b). We also observed some anomalous gene expression patterns associated with SG4, SG5, SG6 and SG7 (Additional files 20, 21: Figure S11, S12), possibly the result of sample differences, Blast tool mismatching or experimental errors. In this study, the bHLH genes of SG6 and SG7 had their highest expression levels in shoot and leaves (Fig. 5b). Although PLEXdb did not contain expression data from shoots, most bHLH genes of SG6 and SG7 showed their highest expressions in leaves at the seedling stage. According to the bHLH gene expression data (Fig. 5), we selected six bHLH genes and assayed their expression by qPCR. Most of these genes displayed expression patterns identical to those revealed from bioinformatics analysis of anthesis-period seeds. These results indicate that bHLH TFs with similar structures may have similar functions. bHLH TFs in different subfamilies that have identical expression patterns may participate in the same network to cooperatively regulate plant processes.

## Conclusion

Since the importance for development and stress tolerance, 225 bHLH TFs predicted from wheat were subjected to identification, classification, phylogenetic reconstruction, conserved motif characterization, chromosomal distribution determination and expression pattern analysis. The bHLH proteins could be separated into four categories based on sequences in their basic regions. Neighbor-joining-based phylogenetic analysis of conserved bHLH domains from wheat, *Arabidopsis* and rice identified 26 subfamilies of bHLH TFs, of which 23 were found in wheat. A total of 82 wheat bHLH genes had orthologs in *Arabidopsis* (27 TFs), rice (28 TFs) and both of them (27 TFs). Seven tissue-specific bHLH TF clusters were identified according to their expression patterns in endosperm, aleurone, seedlings, heading-stage spikes, flag leaves, shoots and roots. Expression levels of six endosperm-specifically expressed TFs measured by qPCR and RNA-seq showed a good correlation. This comprehensive and systematic analysis of bHLH transcription factors in wheat reveals that those TFs from the same subfamily with similar sequence motifs generally have similar expression patterns. The findings in this work provided useful information for functional analysis of bHLH that could be used for improving of agronomic and economic benefit in wheat.

## Additional files

Additional file 1: Table S1. Reference genes of bHLH genes expression analysis, the genes were downloaded from the database of NCBI. (XLSX 25 kb)

Additional file 2: Table S2. RNA-seq information of different tissues or organs at different growth period. (XLSX 15 kb)

Additional file 3: Table S3. The gene expression levels for wheat bHLH transcription factor family at different growth periods in different tissues and organs. (XLSX 197 kb)

Additional file 4: Table S4. The specific primers of bHLH gene sequences in wheat. (XLSX 118 kb)

Additional file 5: Table S5. Two hundred twenty-five bHLH transcription factor nucleotide sequence of wheat. (XLSX 39 kb)

Additional file 6: Table S6. Two hundred twenty-five bHLH transcription factor protein sequence of wheat. (XLSX 29 kb)

Additional file 7: Figure S1. A Venn diagram showing the intersections of bHLH TFs that from plantTFDB, wheatTFDB and this study. The data of plantTFDB, wheatTFDB and this study marked by a, b, and c respectively. The diagram of bHLH gene indicate that bHLH TFs from our research may correspond to multiple bHLH TFs of plantTFDB and wheatTFDB in the Intersections. (PDF 823 kb)

Additional file 8: Figure S2. Multiple sequence alignment of the 225 wbHLH TF amino acid sequences. Shown at the top are the boundaries used in this study to distinguish the DNA-binding basic region, the two a-helixes and the variable loop region. (PDF 1236 kb)

Additional file 9: Figure S3. The neighbor-joining (NJ) phylogenetic tree and conserved motif compositions of wheat (lift). The NJ tree of wheat bHLH genes and their motif locations (right). Logos of wheat bHLH proteins motifs by MEME. Logos are a visualization tool for motifs. The height of a letter indicates its relative frequency at the given position. Fifteen conserved motifs were identified and named motif 1 through motif 15. Two hundred twenty-five bHLH genes were grouped to 24 subfamilies according to the gene structure. (PDF 15106 kb)

Additional file 10: Table S7. Location of wheat bHLH on chromosome. (XLSX 16 kb)

Additional file 11: Table S8. Distribution of the identified bHLH TF genes on wheat chromosomes. (XLSX 15 kb)

Additional file 12: Table S9. Orthologous genes of wbHLH to Arabisopsis and rice. (XLSX 18 kb)

Additional file 13: Figure S4. The network of 20 bHLH TFs that significant homologues to Arabisopsis proteins. The protein-protein interactions was identified in STRING database. (PDF 497 kb)

Additional file 14: Figure S5. The KEGG pathway of wbHLH006 (PIF3) and wbHLH095 (MYC2) involved in plant hormone signal transduction pathway from *Arabidopsis*. (PNG 46 kb)

Additional file 15: Figure S6. The KEGG pathway of wbHLH026 (PIF3) involved in circadian rhythm pathway from *Arabidopsis*. (PNG 19 kb)

Additional file 16: Figure S7. The KEGG pathway of wbHLH006 (PIF3) and wbHLH095 (MYC2) involved in plant hormone signal transduction pathway from sorghum. (PNG 31 kb)

Additional file 17: Figure S8. The KEGG pathway of wbHLH026 (PIF3) involved in circadian rhythm pathway from sorghum. (PNG 19 kb)

Additional file 18: Figure S9. The KEGG pathway of wbHLH006 (PIF3) and wbHLH095 (MYC2) involved in plant hormone signal transduction pathway from maize. (PNG 31 kb)

Additional file 19: Figure S10. The KEGG pathway of wbHLH026 (PIF3) involved in circadian rhythm pathway from maize. (PNG 19 kb)

Additional file 20: Figure S11. The wbHLH genes of block b showed higher expression levels in anthers at before anthesis stage. We obtained the result by blast tool that we used gene sequences mapped the 61 k wheat GeneChip in PLEXdb. The bHLH TFs of black b and gene probe were marked by the labels under each chart, respectively. (JPEG 2270 kb)

Additional file 21: Figure S12. The wbHLH genes of block c showed higher expression levels in leaves at seedling stage. We obtained the result by blast tool that we used gene sequences mapped the 61 k wheat GeneChip in PLEXdb. The bHLH TFs of black c and gene probe were marked by the labels under each chart, respectively. (JPEG 2324 kb)

Additional file 22: Table S10. Correlation analysis of wbHLH genes expression pattern based on RNA-seq data and Q-PCR analysis. (XLSX 54 kb)

Additional file 23: Table S11. Compared plantTFDB, wheatTFDB and this study. (XLSX 8 kb)

Additional file 24: Table S12. The homeologous gene of bHLH in wheat. The wbHLH TFs that lost homologous genes on one of A, B or D genome marked with orange background and lost homologous genes on two genomes marked with green background. (XLSX 12 kb)

## Abbreviations

bHLH: basic helix-loop-helix
DPA: Days post-anthesis
FPKM: Fragments per kilobase of transcript per million fragments mapped
HAI: Hours after imbibition
KEGG: Kyoto encyclopedia of genes and genomes
TFs: Transcription factors
wbHLH: wheat basic helix-loop-helix
