# Supplementary figures and images for: Global identification, structural analysis and expression characterization of bHLH transcription factors in wheat
Source: BMC Plant Biol. 2017 May 30;17:90. doi: 10.1186/s12870-017-1038-y (PMC5450219; doi:10.1186/s12870-017-1038-y)

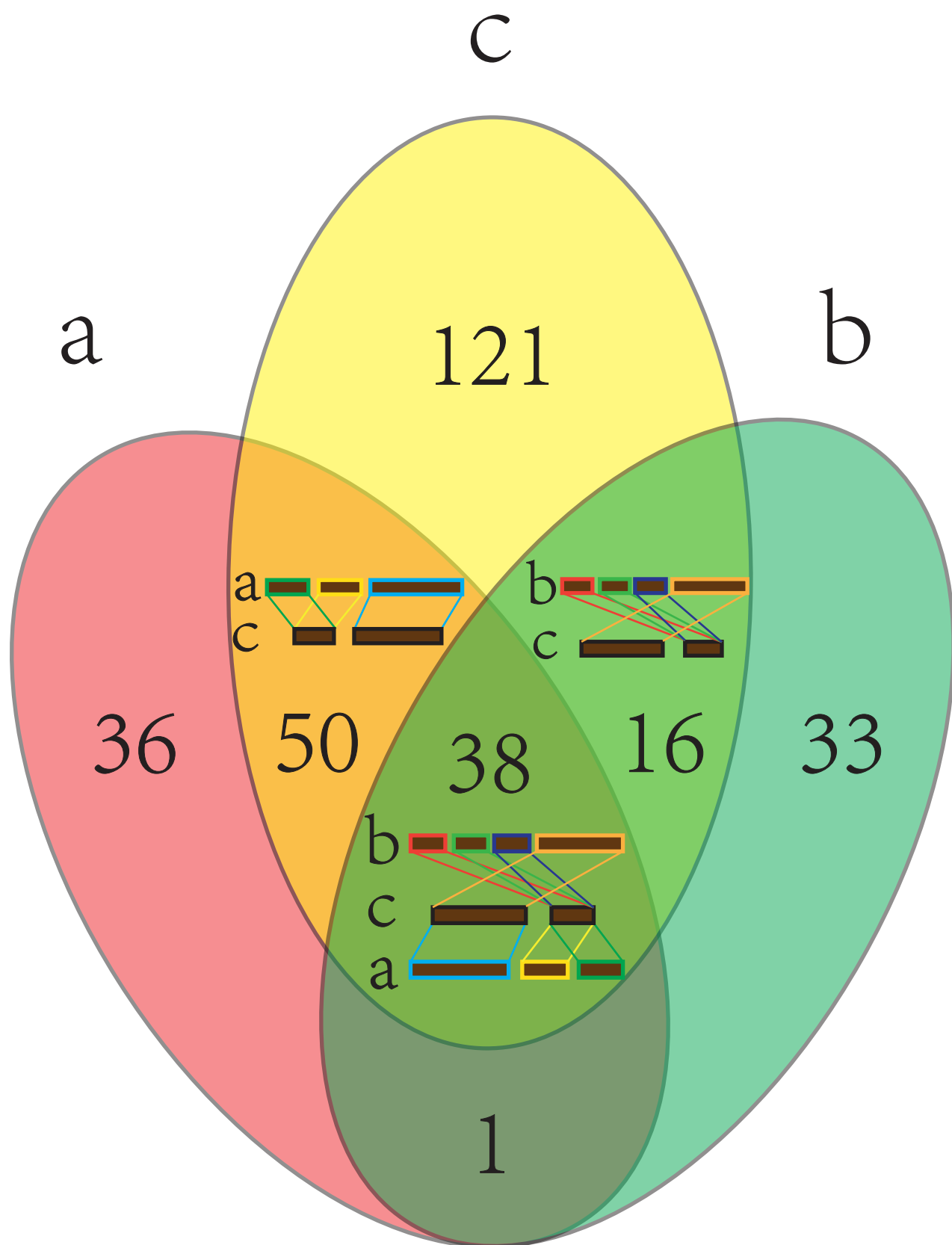

Supplement: Supplementary file 7 — A Venn diagram showing the intersections of bHLH TFs that from plantTFDB, wheatTFDB and this study. The data of plantTFDB, wheatTFDB and this study marked by a, b, and c respectively. The diagram of bHLH gene indicate that bHLH TFs from our research may correspond to multiple bHLH TFs of plantTFDB and wheatTFDB in the Intersections. (PDF 823 kb) [file 12870_2017_1038_MOESM7_ESM.pdf]

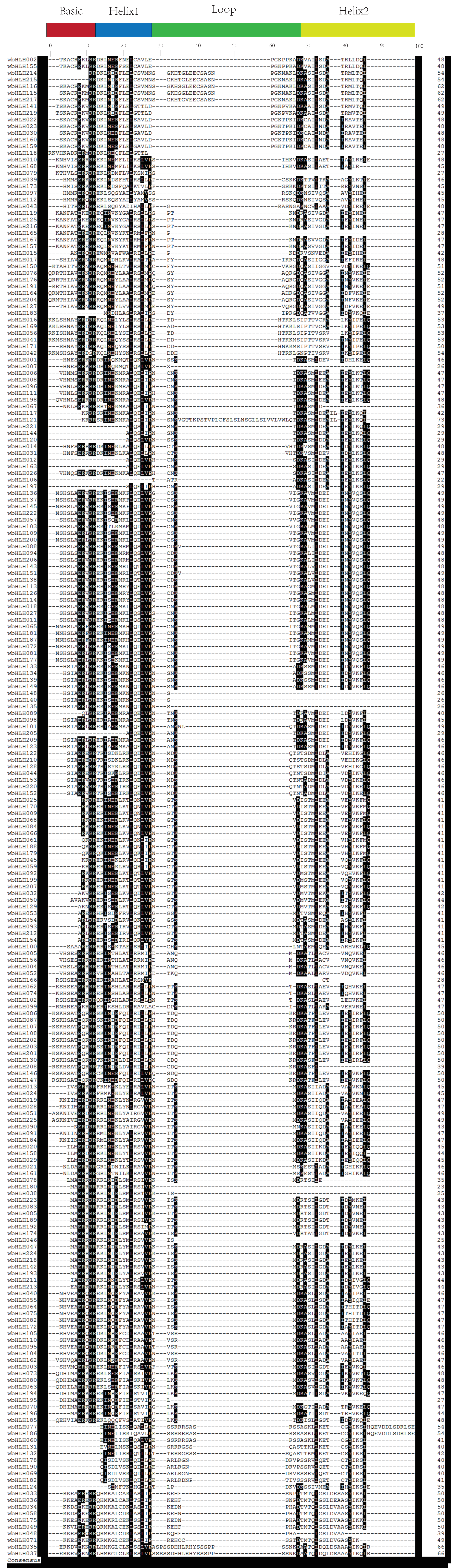

Supplement: Supplementary file 8 — Multiple sequence alignment of the 225 wbHLH TF amino acid sequences. Shown at the top are the boundaries used in this study to distinguish the DNA-binding basic region, the two a-helixes and the variable loop region. (PDF 1236 kb) [file 12870_2017_1038_MOESM8_ESM.pdf]

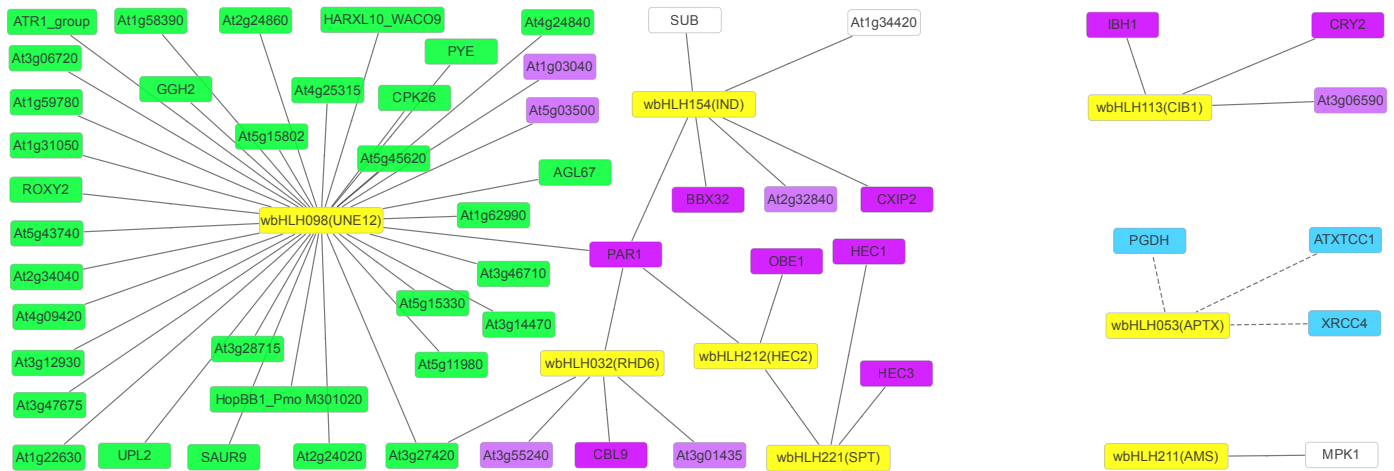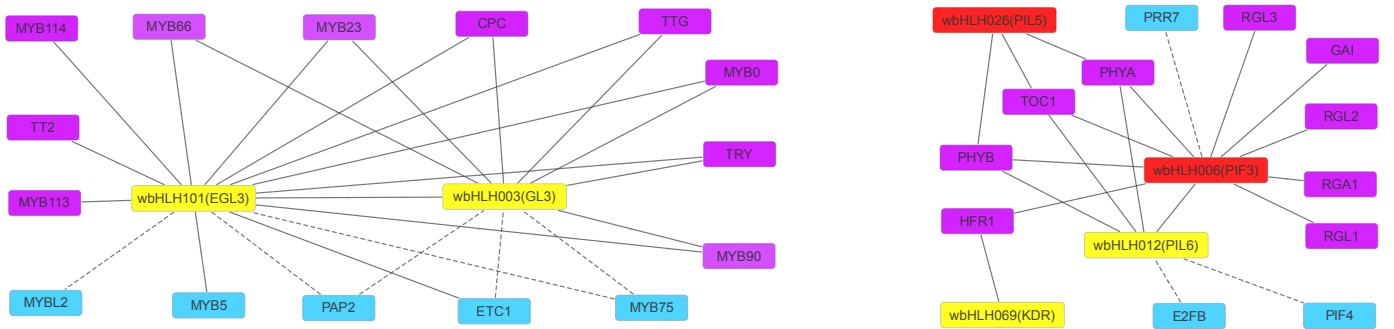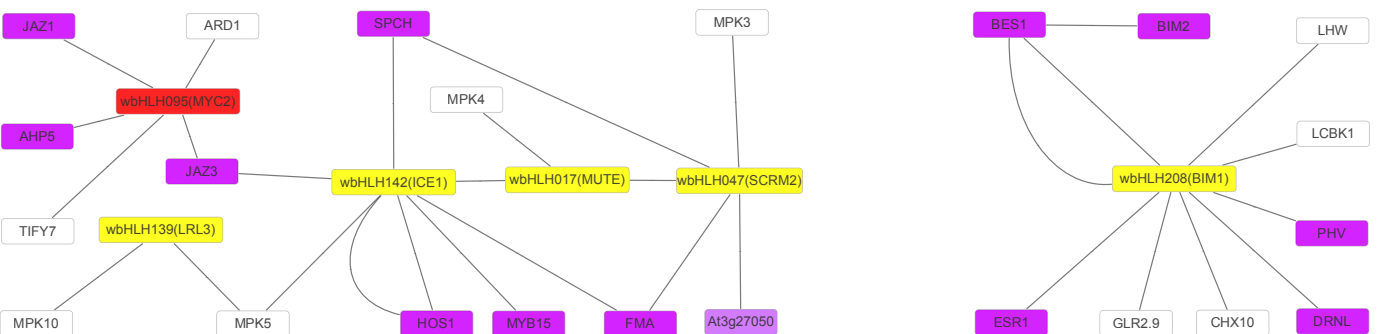

Supplement: Supplementary file 13 — The network of 20 bHLH TFs that significant homologues to Arabisopsis proteins. The protein-protein interactions was identified in STRING database. (PDF 497 kb) [file 12870_2017_1038_MOESM13_ESM.pdf]

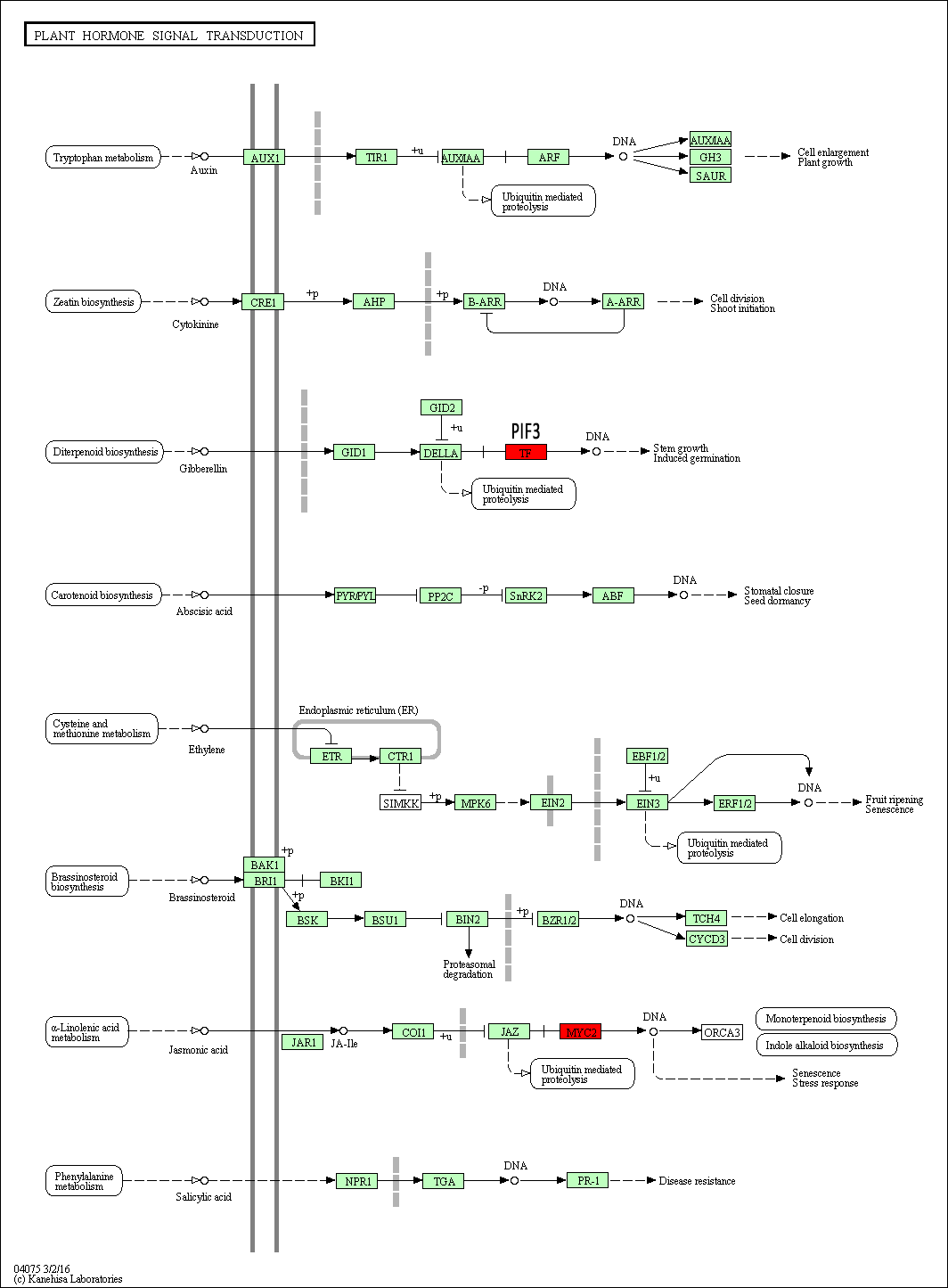

Supplement: Supplementary file 14 — The KEGG pathway of wbHLH006 (PIF3) and wbHLH095 (MYC2) involved in plant hormone signal transduction pathway from Arabidopsis. (PNG 46 kb) [file 12870_2017_1038_MOESM14_ESM.png]

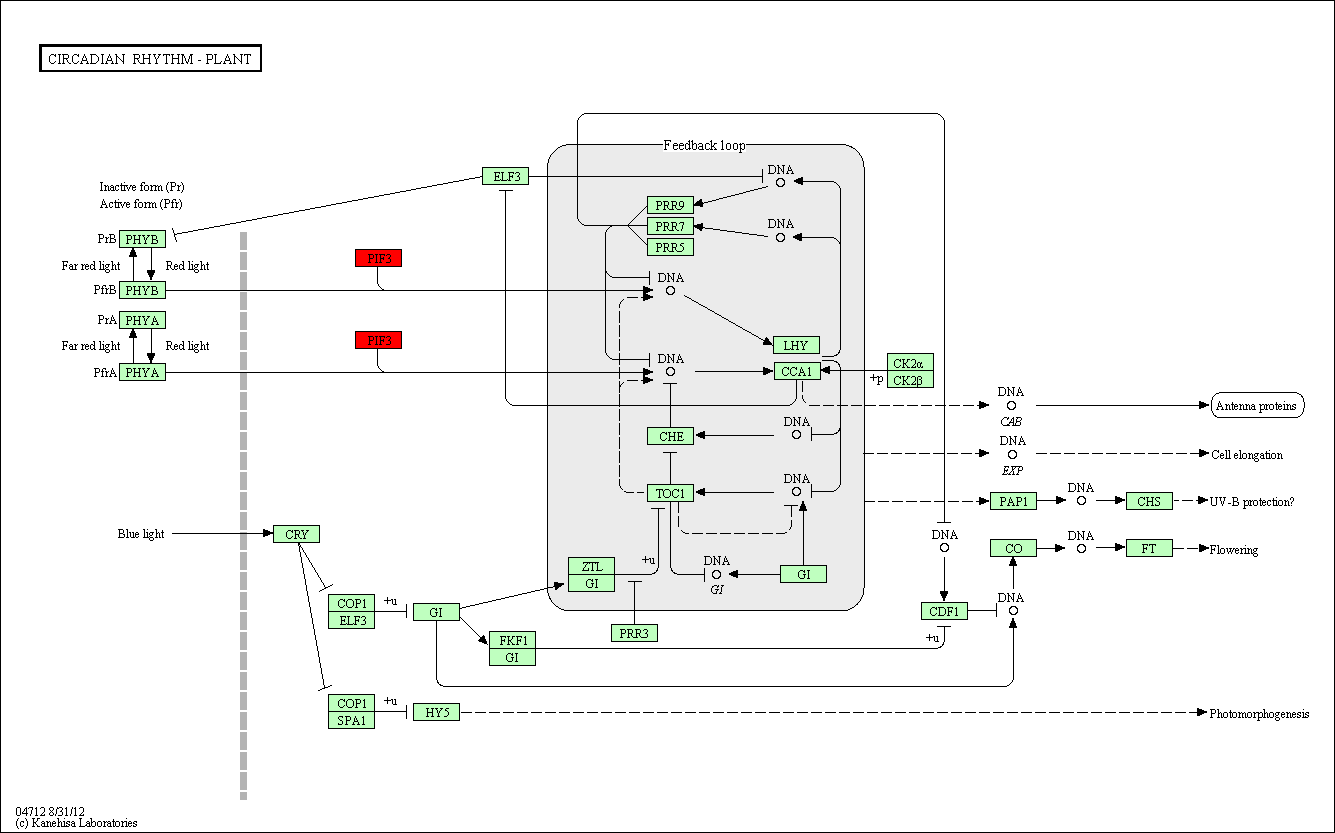

Supplement: Supplementary file 15 — The KEGG pathway of wbHLH026 (PIF3) involved in circadian rhythm pathway from Arabidopsis. (PNG 19 kb) [file 12870_2017_1038_MOESM15_ESM.png]

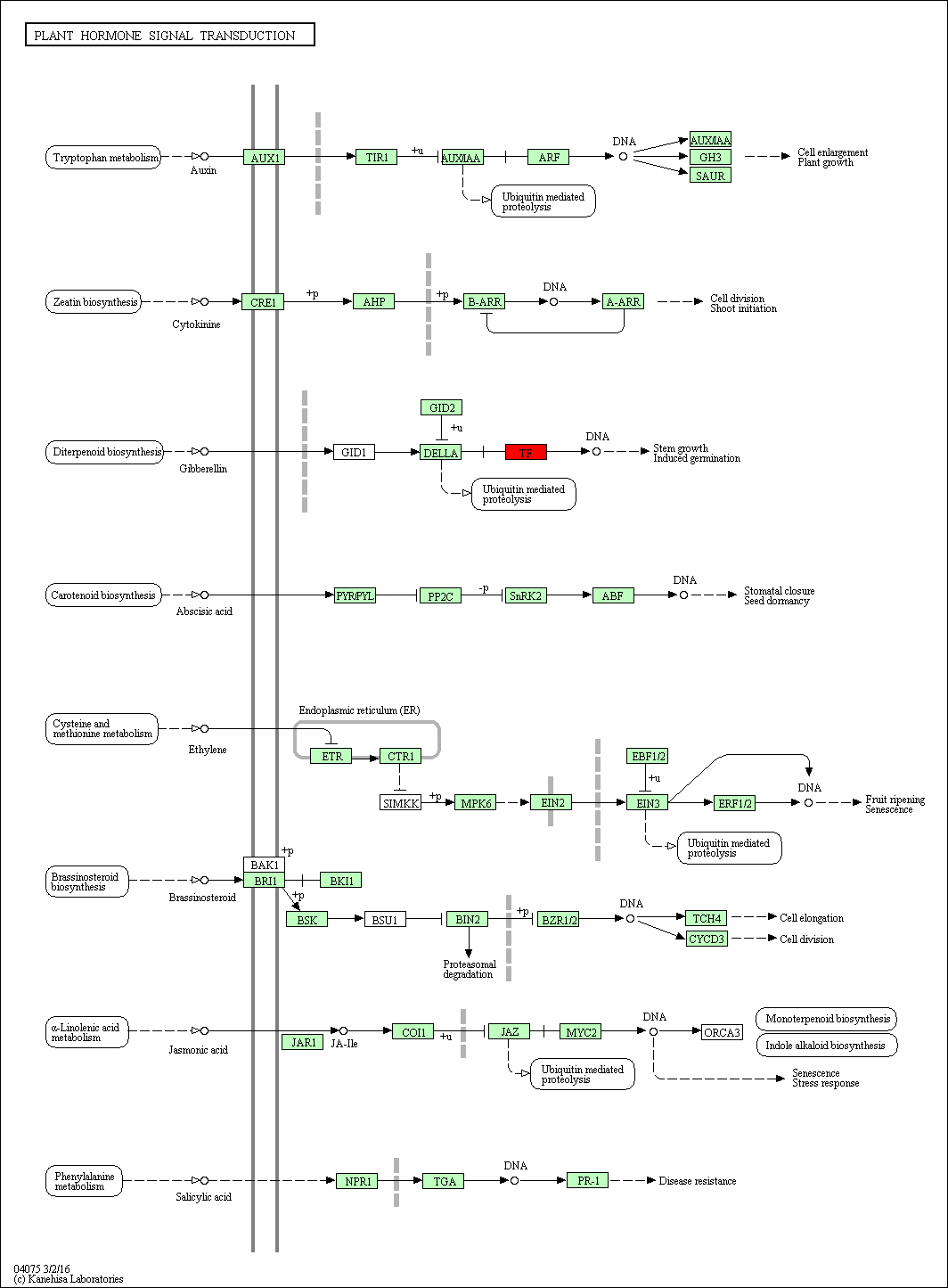

Supplement: Supplementary file 16 — The KEGG pathway of wbHLH006 (PIF3) and wbHLH095 (MYC2) involved in plant hormone signal transduction pathway from sorghum. (PNG 31 kb) [file 12870_2017_1038_MOESM16_ESM.png]

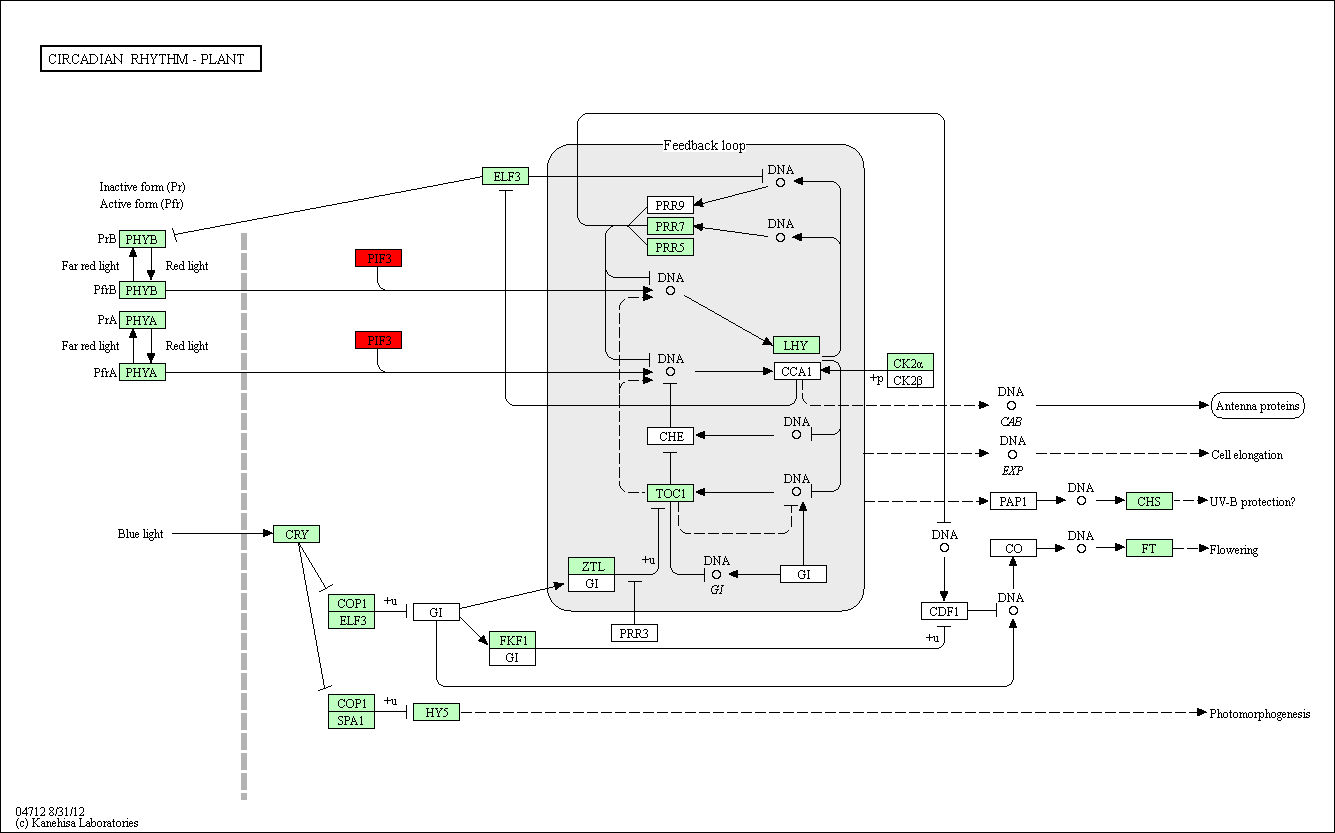

Supplement: Supplementary file 17 — The KEGG pathway of wbHLH026 (PIF3) involved in circadian rhythm pathway from sorghum. (PNG 19 kb) [file 12870_2017_1038_MOESM17_ESM.png]

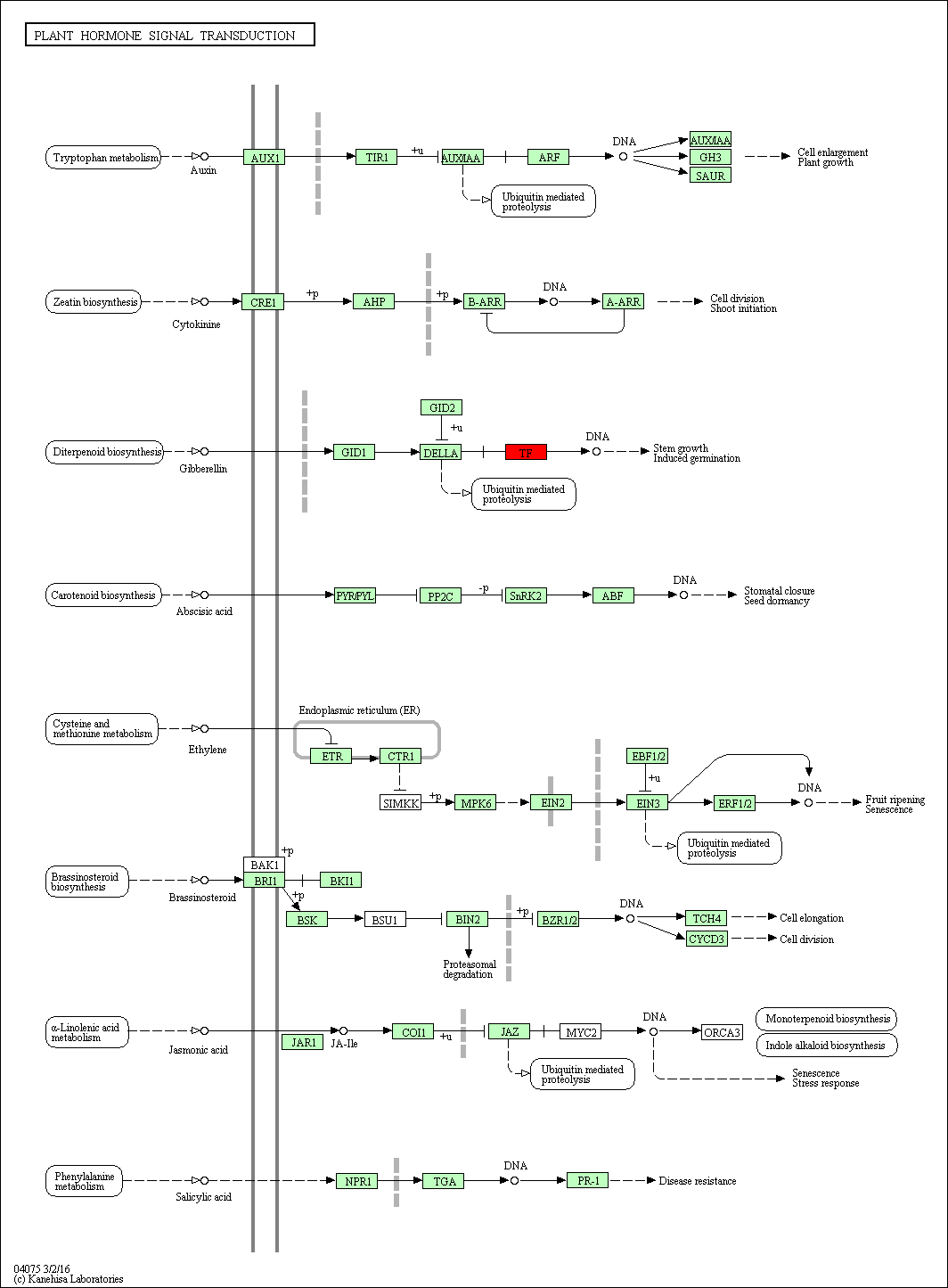

Supplement: Supplementary file 18 — The KEGG pathway of wbHLH006 (PIF3) and wbHLH095 (MYC2) involved in plant hormone signal transduction pathway from maize. (PNG 31 kb) [file 12870_2017_1038_MOESM18_ESM.png]

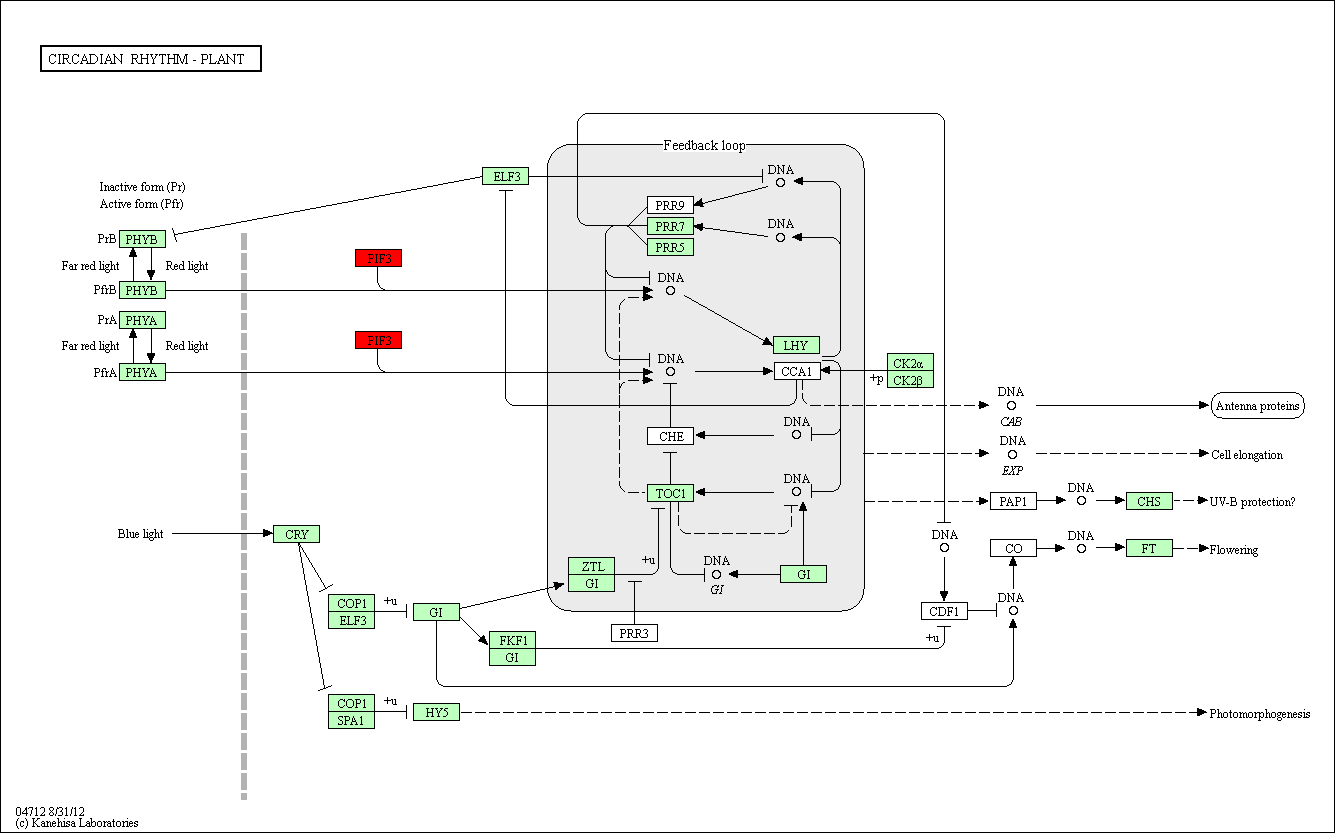

Supplement: Supplementary file 19 — The KEGG pathway of wbHLH026 (PIF3) involved in circadian rhythm pathway from maize. (PNG 19 kb) [file 12870_2017_1038_MOESM19_ESM.png]

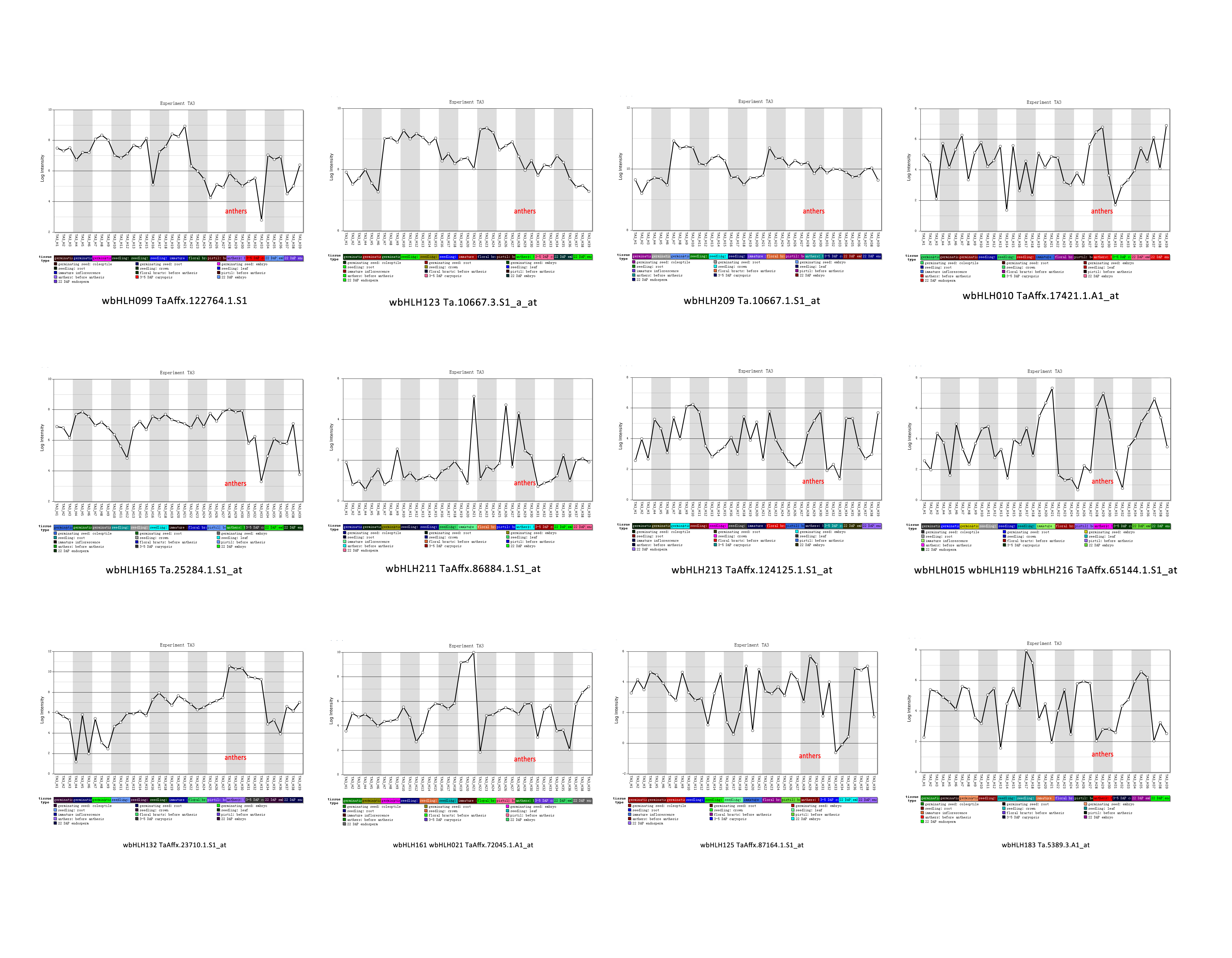

Supplement: Supplementary file 20 — The wbHLH genes of block b showed higher expression levels in anthers at before anthesis stage. We obtained the result by blast tool that we used gene sequences mapped the 61 k wheat GeneChip in PLEXdb. The bHLH TFs of black b and gene probe were marked by the labels under each chart, respectively. (JPEG 2270 kb) [file 12870_2017_1038_MOESM20_ESM.jpg]

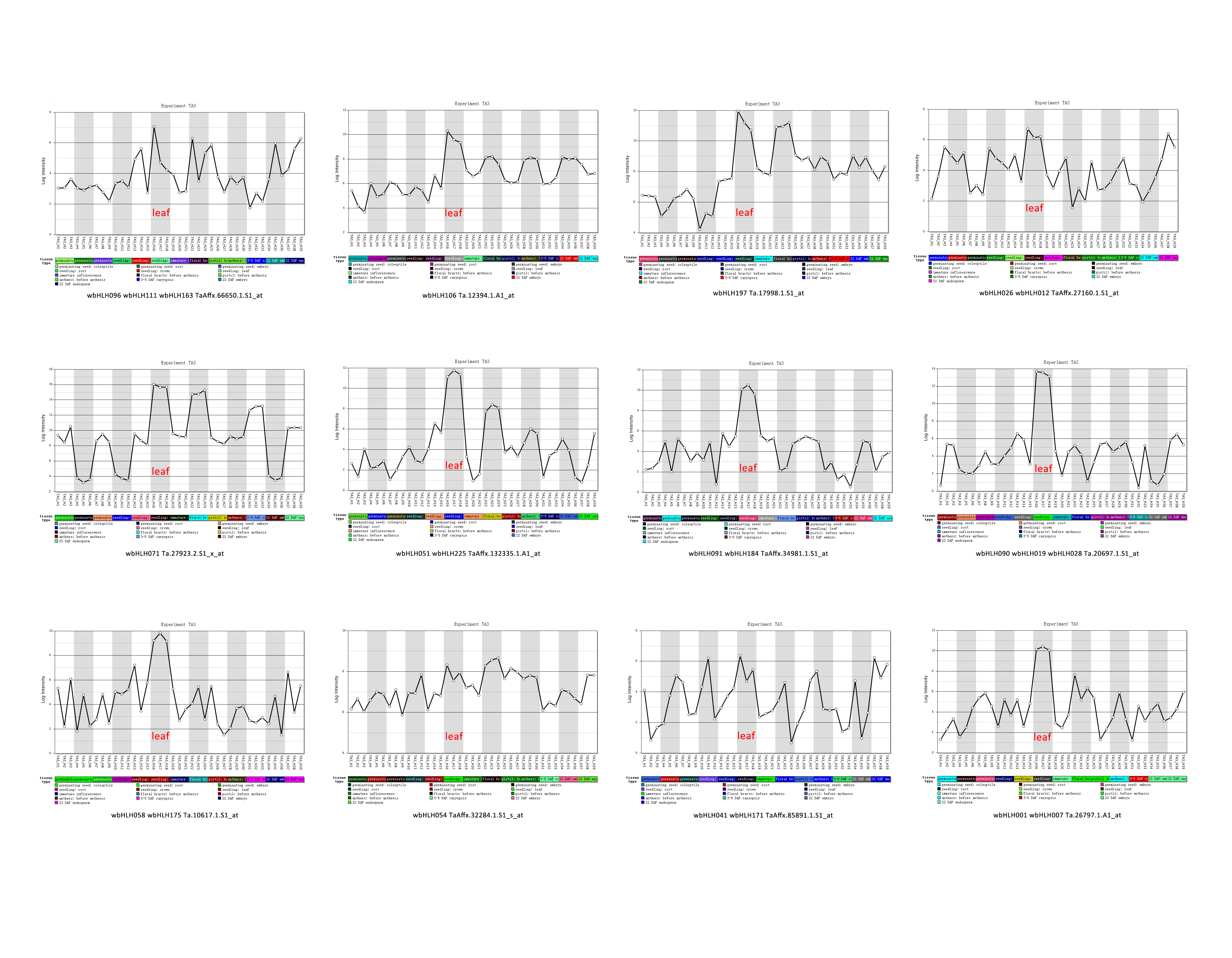

Supplement: Supplementary file 21 — The wbHLH genes of block c showed higher expression levels in leaves at seedling stage. We obtained the result by blast tool that we used gene sequences mapped the 61 k wheat GeneChip in PLEXdb. The bHLH TFs of black c and gene probe were marked by the labels under each chart, respectively. (JPEG 2324 kb) [file 12870_2017_1038_MOESM21_ESM.jpg]
