# Supplementary material for: Global identification, structural analysis and expression characterization of bHLH transcription factors in wheat
Source: BMC Plant Biol. 2017 May 30;17:90. doi: 10.1186/s12870-017-1038-y (PMC5450219; doi:10.1186/s12870-017-1038-y)
Supplement: Supplementary file 9 — The neighbor-joining (NJ) phylogenetic tree and conserved motif compositions of wheat (lift). The NJ tree of wheat bHLH genes and their motif locations (right). Logos of wheat bHLH proteins motifs by MEME. Logos are a visualization tool for motifs. The height of a letter indicates its relative frequency at the given position. Fifteen conserved motifs were identified and named motif 1 through motif 15. Two hundred twenty-five bHLH genes were grouped to 24 subfamilies according to the gene structure. (PDF 15106 kb) [file 12870_2017_1038_MOESM9_ESM.pdf]

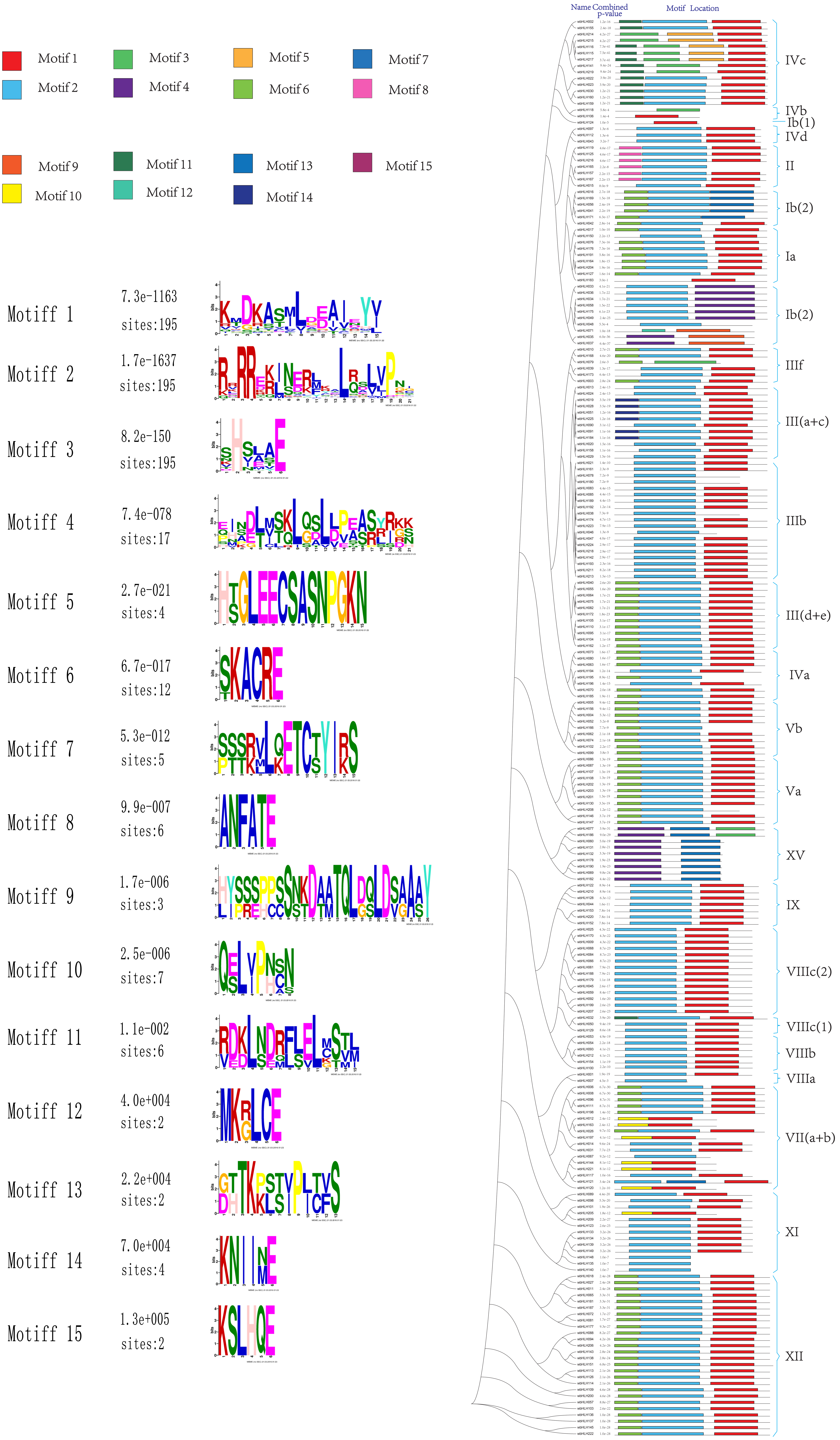
Motif 1
Motif 2
Motif 3
Motif 4
Motif 5
Motif 6
Motif 7
Motif 8
Motif 9
Motif 10
Motif 11
Motif 12
Motif 13
Motif 14
Motif 15
Motiff 1 7.3e-1163 sites:195
Motiff 2 1.7e-1637 sites:195
Motiff 3 8.2e-150 sites:195
Motiff 4 7.4e-078 sites:17
Motiff 5 2.7e-021 sites:4
Motiff 6 6.7e-017 sites:12
Motiff 7 5.3e-012 sites:5
Motiff 8 9.9e-007 sites:6
Motiff 9 1.7e-006 sites:3
Motiff 10 2.5e-006 sites:7
Motiff 11 1.1e-002 sites:6
Motiff 12 4.0e+004 sites:2
Motiff 13 2.2e+004 sites:2
Motiff 14 7.0e+004 sites:4
Motiff 15 1.3e+005 sites:2
Name Combined p-value
Motif Location
IVc
IVb
Ib(1)
IVd
II
Ib(2)
Ia
Ib(2)
IIIf
III(a+c)
IIIb
III(d+e)
IVa
Vb
Va
XV
IX
VIIIc(2)
VIIIc(1)
VIIIb
VIIIa
VII(a+b)
XI
XII
